# Metabolic Improvement via Enhancing Thermogenic Fat-Mediated Non-shivering Thermogenesis: From Rodents to Humans

**DOI:** 10.3389/fendo.2020.00633

**Published:** 2020-09-10

**Authors:** Ruping Pan, Xiaohua Zhu, Pema Maretich, Yong Chen

**Affiliations:** ^1^Department of Nuclear Medicine, Tongji Medical College, Tongji Hospital, Huazhong University of Science and Technology, Wuhan, China; ^2^Department of Biology, Massachusetts Institute of Technology, Cambridge, MA, United States; ^3^Department of Endocrinology, Internal Medicine, Tongji Medical College, Tongji Hospital, Huazhong University of Science and Technology, Wuhan, China

**Keywords:** obesity, brown adipose tissue, beige adipose tissue, non-shivering thermogenesis, human, rodent

## Abstract

Brown and beige adipose tissues play a large role in non-shivering thermogenesis (NST) in mammals, and subsequently have been studied for decades as potential therapeutic targets to treat obesity and its related metabolic diseases. However, the mechanistic regulation of brown/beige adipose tissue induction and maintenance in humans is very limited due to the ethical reasons. In fact, metabolic signaling has primarily been investigated using rodent models. A better understanding of non-shivering thermogenesis in humans is thus vital and urgent in order to treat obesity by targeting human brown adipose tissue (BAT). In this review, we summarize the anatomical and physiological differences between rodent and human BAT, current useful and mostly non-invasive methods in studying human BAT, as well as recent advancements targeting thermogenic adipocytes as a means to combat metabolic diseases in humans. Furthermore, we also discuss several novel relevant strategies of therapeutic interventions, which has been attempted in rodent experiments, and possible future investigations in humans in this field.

## Introduction

In mammals, there are three kinds of adipose tissues participating in whole-body energy homeostasis. They include white adipose tissue (WAT), which stores energy in the form of triglycerides, brown adipose tissue (BAT), which dissipates energy into heat, and beige adipose tissue, which functions similarly to BAT. BAT, first characterized in 1960s, has been described as both an endocrine and a thermogenic organ (1). It consists of morphologically distinct brown adipocytes which contain multilocular lipid droplets and abundant mitochondria. BAT is the main organ which contributes to non-shivering thermogenesis (NST) in mammals (2). Classically, in response to cold, BAT activation is dependent on the sympathetic innervation (involvement of norepinephrine) and the activation of β3-adrenergic receptors (ARs) located mainly on the adipocyte membrane, followed by a lipolysis from stored triglycerides to free fatty acids, which drives mitochondria respiration and is then oxidized during this process (3–5). BAT is also involved in diet-induced thermogenesis (6), which is dependent on local sympathetic innervation and AR signaling as well (7, 8). Furthermore, NST is largely dependent on uncoupling protein 1 (UCP1), a BAT specific protein located on the mitochondrial membrane, which uncouples the respiratory chain of oxidative phosphorylation within mitochondria, leading to an increase in ATP consumption and heat generation (9). NST has been long thought to only exist in hibernating animals and human infants because of the wealth of BAT in their body for generating heat under certain circumstances to keep warm. In fact, BAT is found in almost all mammals including mice, rats, rabbits, sheep, bears, and humans except pigs (10, 11). Studies have been mostly performed using rodent models to investigate mechanisms of NST regulation. Meanwhile, brown-like adipocytes, later termed beige adipocytes, were discovered in subcutaneous WAT in rodents in response to cold stimulus (12, 13). They look morphologically similar to brown adipocytes and contain abundant UCP1-positive mitochondria, which supports their role in NST (12, 14). Besides, their activation is also triggered by a sympathetic innervation, which is similar to brown adipocytes as well (15). As long as functional BAT is detected in 2007 (16) and specifically characterized in adult humans in 2009 using ^18^F-fluorodeoxyglucose Positron Emission Tomography coupled with Computer Tomography (^18^F-FDG PET/CT) (17–19), more and more studies are performed to study BAT activation in humans mostly using PET/CT as well as other non-invasive methods due to ethical reasons. ^18^F-FDG positive adipose tissue in humans is primarily distributed in the cervical, supra-clavicular, supra-adrenal, and para-vertebral regions (16). Significantly, human studies have been performed to identify whether those ^18^F-FDG positive adipose tissues in humans are classic BAT or recruitable beige adipose tissue. There is evidence that both classical brown and beige adipocytes exist in human infant through a corpse study using magnetic resonance imaging (MRI), in addition to histological and biochemical analyses (20). In 2013, through anatomical and transcriptome profiling, it was shown that deeper cervical fat consists of classical brown adipocytes while supra-clavicular fat is composed of both classical brown and recruitable beige adipocytes in adult humans (21, 22). Moreover, global and unbiased genome-wide expression analysis of clonally derived adult human brown adipocytes from the supra-clavicular region indicates a close relationship between human brown adipocytes and mouse beige adipocytes (23). Nevertheless, targeting brown and beige adipose tissue, such as the administration of β3-AR agonists, A_2A_ receptor agonists and other pharmaceuticals, promotes thermogenic fat-mediated NST and becomes feasible therapeutic approaches to increase energy expenditure and potentially treat obesity. Besides, certain natural molecules have also been identified to be involved in the regulation of thermogenic fat activation in humans. However, knowledges on the mechanistic regulation of brown and beige adipose tissue-mediated NST are mostly known from rodent experiments, and human BAT is more heterogeneous than rodent BAT due to its composition and possible distinct mRNA-expression profiles (23). Thus, a better understanding of the roles of brown and beige adipose tissue in energy metabolism in humans could provide additional resources to clinically treat obesity and its comorbidities.

## Anatomical and Physiological Differences Between Human and Rodent Bat

**Figure 1:**
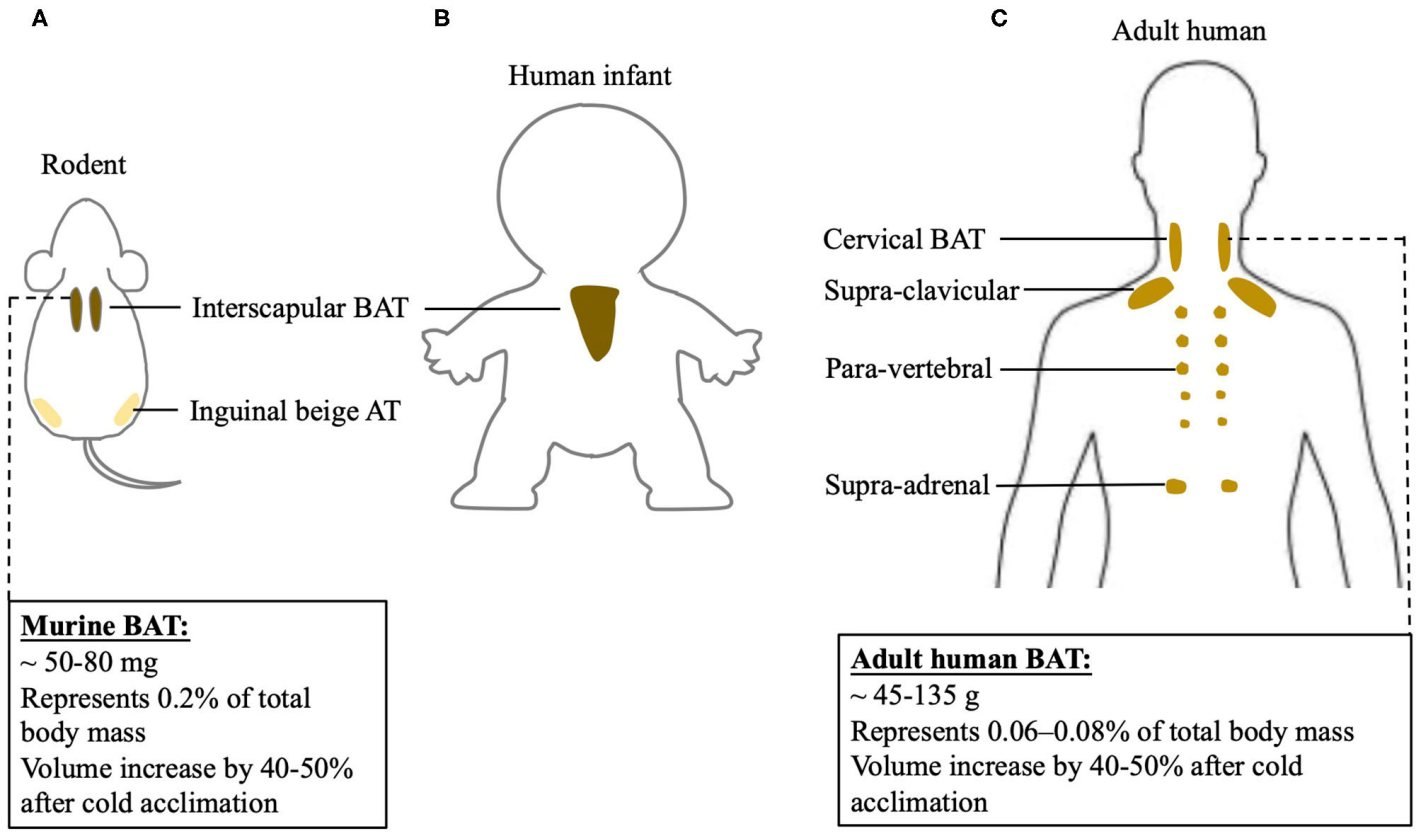
BAT localization in rodent and human and differences of BAT physiology between two species. BAT localization in **(A)** rodent; **(B)** human infant, and **(C)** adult human. Physiological characterization of BAT in mouse and adult human.

An understanding of differences between rodent and human BAT could be of advantage to realize the transition of scientific research achievements from rodent to human. However, the function of BAT and its contribution to energy metabolism in humans may differ from results found in rodents. This discrepancy could be largely due to the anatomical and physiological differences between two species, shown in Figure 1. In rodents, classic BAT exists past the neonatal period into adulthood, while in humans this is still controversial. In human infants, classic BAT is found in the subcutaneous fat depot of interscapular region, and a layer of connective tissue between WAT and BAT is identified histologically (20). However, the ^18^F-FDG PET/CT scans reveal that adult humans do not exhibit interscapular BAT. Nevertheless, the age at which interscapular BAT atrophies and disappears in humans is still unclear. Inspiringly, it has been indicated that in certain individuals, deeper cervical adipose tissue in adult humans shares many similarities with classical rodent BAT on molecular and histological level (21). Moreover, it has been shown that tissue in the supra-clavicular region in adult humans is composed of a mixture of brown and beige adipocytes (22). Thus, adult human BAT is special and unique compared to classic BAT existing in rodents. Whether targeting adult human BAT is adequate for heat generation under certain circumstances still need to be further investigated.

Increasing BAT mass and BAT activity could be potential mechanistic targets to induce an upregulation of BAT-mediated NST. BAT mass can be quantified using PET/CT (24). Specifically, ^18^F-FDG positive adipose tissue with an SUV mean threshold ≥ 1.5 is considered BAT. BAT volume, when multiplied by the density of the tissue, can be used to approximate total BAT mass in an individual (25). Human BAT reported so far is ~45–135 grams, while mice have about 50–80 milligrams of BAT. When calculated as percentage in body weight, human BAT represents 0.06–0.08% of total body mass, while mouse BAT is about 0.2% of total body mass. Cold acclimation in humans increases BAT volume by 40–50% (26–28), an increase similar to what has been observed in mice (29). Using direct PET/CT scan with [^15^O]O_2_ and [^15^O]H_2_O, it has been shown that a short-time mild cold exposure could cause a BAT-mediated oxygen consumption, which is as 0.1–0.6% of whole-body oxygen consumption in humans (30–33). Following a chronic cold exposure for 4 weeks, the contribution of BAT to whole-body oxygen consumption in humans further increases to 0.5–2.3% (24, 34). However, in mice, their oxygen consumption is increased by 38–60% after a mild cold exposure (35, 36). The drastic differences in thermogenic responses to cold stimulation between humans and rodents may be, in part, due to disparities in proportions of BAT mass relative to whole body mass and diverse analysis methods. Moreover, humans may differ from rodents in the mechanisms involved in BAT-induced energy expenditure, which actually remain largely unknown in humans. Thus, the physiological difference between rodent and human BAT has to be noticed when targeting BAT to combat obesity when using rodent models.

## Non-Invasive Methods in Studying Human Bat

^18^F-FDG PET/CT, as a non-invasive method, is commonly used to study human BAT. Active BAT takes glucose as the source of energy metabolism, thus, ^18^F-labled glucose analogue FDG works as a tracer for BAT imaging. When merged with CT images, tracer aggregation in the adipose tissue region could display the location and glucose uptake of BAT. Besides, dynamic metabolic imaging can be obtained after ^18^F-FDG PET/CT scanning, which directly reflects the activity of BAT. However, ^18^F-FDG PET/CT is radioactive, which is harmful and may limit the use of its application. Other non-invasive methods to study human BAT include MRI, infrared thermography (IRT), and orthogonal assays assessing metabolic changes associated with BAT activation, such as whole-body calorimetry. Furthermore, researchers often take BAT biopsies for molecular analysis. The differences between these methods are shown in Table 1.

**Table 1:** Comparison of different methods in studying human BAT.

**Methods**	**Invasion**	**Advantages**	**Disadvantages**
Biopsy (molecular analysis)	Minor invasion	Data on molecular level	Low accuracy of sampling
PET/CT	Non-invasion	Dynamic metabolic imaging	Radiation
MRI	Non-invasion	Non-radiation	Complex modeling, low sensitivity
IRT	Non-invasion	Non-radiation	Difficult anatomical localization
Calorimetry	Non-invasion	Assistant method	Low application value when used alone
Blood testing (certain biomarkers)	Non-invasion	Easy operation	Needs further validation

MRI can be used to assess the intracellular triglyceride depletion of human BAT by measuring fat content before and after BAT activation (37). Unlike PET/CT, MRI does not require radiation. However, due to its complex modeling and low sensitivity, it is not applied as frequently as PET/CT to quantify human BAT.

Similar to the MRI, IRT does not require radioactivity for its measurements. The anterior supraclavicular temperature measured by IRT has been shown to be positively correlated with energy expenditure and changes in parallel with standard uptake value (SUV) obtained from PET imaging (38, 39). Nevertheless, IRT is mostly useful for measuring the temperature of superficial adipose tissue, which might be inapplicable and imprecise for a temperature measurement of deeper parts. Besides, the anatomical localization of an IRT scan appears to be difficult.

Whole-body calorimetry can be used to determine energy expenditure of humans (38). When paired with blood serum analysis of metabolites such as high-density lipoprotein, triglycerides, fasting glucose, non-esterified fatty acids, etc., this method provides insight into whole-body energy metabolism. It is often paired with other methods of studying BAT function in humans.

Remarkably, certain biomarkers in serum have been characterized in several studies that correlates with BAT mass and BAT activity in humans. A previous study from our lab revealed that serum concentration of miRNA-92a, derived from BAT exosomes, is negatively correlated with human BAT activity (40). Similarly, BAT-derived exosomal miR-122-5p, has also been shown to be negatively correlated with human BAT activity (41). Meanwhile, a recent study reported a positive correlation of lysophosphatidylcholine-acyl C16:0 and Fibroblast growth factor 21 (FGF21) with human BAT activity (42, 43). Undoubtedly, novel diagnostic tools are needed for assessing BAT function in large and repeated cohort studies in humans.

## Current Advancements of Combating Obesity With Bat in Humans

**Figure 2:**
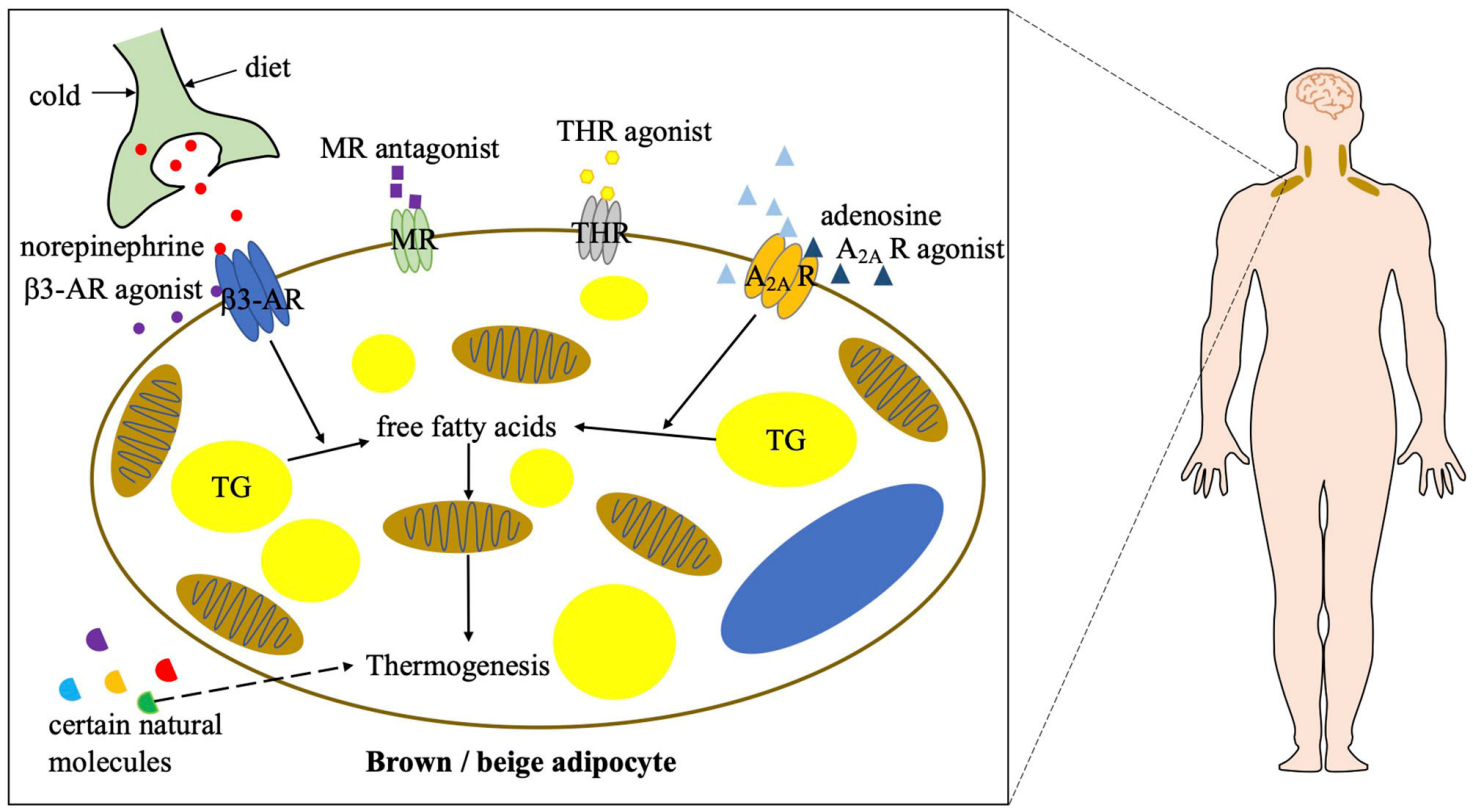
Current strategies of combating obesity via targeting human BAT. A_2A_ R, A_2A_ receptor; β3-AR, beta 3 adrenergic receptor; MR, mineralocorticoid receptor; THR, thyroid hormone receptor; TG, triglycerol.

Targeting brown and beige adipose tissue has been a viable therapeutic approach to combat obesity. The role of these two types of thermogenic adipose tissue is better established in rodents, but their function and regulation in adult humans remain largely unknown. Although cold exposure is an effective way to stimulate BAT activity in humans, pharmacological stimulations are much more achievable and efficient. Based on the findings in rodent experiments that the β3-AR signaling and adenosine–A_2A_ receptor signaling play prominent roles in the regulation of BAT function (7, 44, 45), the effects of β3-AR agonists and adenosine on BAT activation and energy metabolism have been studied in humans (Figure 2).

### β3-AR Agonists

In the last few decades, different β3-AR agonists were developed by companies and their contribution to BAT activation has been studied. The effects of β3-AR agonists on thermogenic fat-mediated energy metabolism have long been observed in rodents. However, due to a lower expression of β3-AR in human adipocytes compared with murine adipocytes, most β3-AR agonists have poor bioavailability in patients (46). On the other hand, due to a low selectivity of those β3-AR agonists and localization of β3-AR elsewhere, they can have fatal effects on the cardiovascular system (46–48). None of the previous β3-AR agonists have been approved for clinical use to treat metabolic diseases. In recent years, several β3-AR agonists including mirabegron, vibegron, ritobegron, and solabegron have been repurposed for other diseases (49–51). Some have been approved for clinical use to treat overactive bladders and urinary incontinence. Their effects on BAT activation and metabolism in humans have been the focus of several clinical trials as well. The results show that both acute (2 days) and chronic (28 days) administration of mirabegron dramatically induces BAT activity, measured by PET/CT, and boosts resting energy expenditure in healthy humans (52, 53). Moreover, biomarkers indicative of healthy metabolism such as high-density lipoprotein, ApoA1, non-esterified fatty acids, total bile acids and adiponectin are increased, and insulin sensitivity is improved after mirabegron treatment. Furthermore, in obese and insulin-resistant humans, chronic mirabegron administration promotes glucose tolerance and induces “beiging” in subcutaneous WAT, in parallel with an improvement of β-cell function (54). However, mirabegron induced metabolic upregulation does not result in weight loss. Despite of an accelerated heartbeat and increased systolic blood pressure after mirabegron treatment (53), mirabegron administration may promote BAT activity and thereby benefits obesity and obesity-related metabolic disorders. Further studies are needed to develop novel applications of β3-AR agonists and, in particular, reduce the above by-effects to treat metabolic diseases.

### Adenosine and A_2A_ Receptor Agonists

Adenosine is an extracellular molecule involved in whole-body energy metabolism. In response to sympathetic stimulation by noradrenaline, an endogenous adenosine is released locally in BAT (44). Adenosine in binding with A_2A_ receptors has been shown to not only increase BAT activation but also induce “beiging” in rodents, resulting in a reduction in diet-induced obesity and an improvement in glucose tolerance. Furthermore, an A_2A_ receptor agonist, CGS21680, also induces BAT activation and results in an increase in energy expenditure in mice. The effect of exogenous adenosine on human BAT as well as A_2A_ receptor density has been investigated using PET/CT imaging (55). It has been shown that adenosine administration dramatically increases BAT activity in humans. Its induced BAT activation is even greater than that induced by cold exposure. Besides, radioligand detectable A_2A_ receptors decrease after cold exposure due to a release of endogenous adenosine, which binds on the A_2A_ receptors. Collectively, these results indicate that targeting A_2A_ receptors on thermogenic adipocytes is potentially another approach to treat obesity. Specifically, adenosine and A_2A_ receptor agonists could be potential therapeutic drugs to enhance BAT function. However, further investigations are required to assess their safety, considering their potentially deleterious effects on the cardiovascular system.

### Other Potential Approaches to Combat Obesity With Human BAT

Other well-known pharmacological approaches to stimulate human BAT activity also include PPARγ agonists, mineralocorticoid receptor antagonists, and thyroid hormone receptor agonists. Among them, certain PPARγ agonists have been shown to potentially induce beige fat development (56, 57), which may be beneficial in the treatment of obesity and its related metabolic disorders. Mineralocorticoid receptor antagonists have been shown to positively correlates to BAT thermogenesis in humans (58), which may also potentially benefit obesity. Thyroid hormones have been long discovered to induce thermogenesis and subsequent high metabolic rate in humans, which is thought to be caused through a mechanism involving the activation of human BAT. Certain thyroid hormone receptor agonists have been identified to promote beige fat development and induce heat generation in rodents even at ambient temperature (59). However, the mechanisms of the above pharmaceuticals in thermogenic fat activation remain unclear, so that their roles in human BAT activation and obesity treatment need to be further investigated. In addition to pharmacological approaches, some natural molecules are also involved in human BAT activation, which includes secretin, cardiac natriuretic peptides, bile acids, myokines, capsaicin, and so on (60–64). They may also contribute to BAT-mediated energy consumption and benefit obese patients. However, their individual mechanisms are still disputed or frankly unclear. Further investigations of these molecules in energy metabolism in humans are required.

## Prospects From Rodent Experiments to a Better Metabolic Health in Humans

There is increasing evidence that BAT acts physiologically as a “metabolic sink” in the human body (65). ^18^F-FDG and ^18^F-fluoro-thiaheptadecanoic acid (^18^F-FTHA) PET/CT imaging clearly display a dynamic uptake of glucose and free fatty acids into the BAT after cold stimulation (66). BAT plays an important role in glucose homeostasis and promoting insulin sensitivity in humans (67). Its oxidative capacity is largely associated with whole body energy expenditure. At thermoneutrality, food intake activates glucose uptake in human BAT via diet-induced thermogenesis (6). Furthermore, BAT has been shown to contribute to excessive energy expenditure under certain pathological conditions such as hyperthyroidism and cachexia, which are both characterized by emaciation (68, 69). Of note, human BAT is different to murine BAT due to its composition, localization, and oxidative capacity after certain stimulation. An anatomical and physiological comparison of BAT between human and rodent mentioned in previous paragraph may be inadequate to conclude the difference of human and rodent BAT contribution to whole body energy metabolism. However, these shortcomings should be taken into consideration when using rodent models to study BAT.

Gene profiling of human ^18^F-FDG positive adipose tissues indicates a cellular heterogeneity of adult human BAT (21–23), which is still being investigated. It is known that thermogenic adipocytes respond to cold and pharmacological stimulation (52, 53, 55), which is similar to the findings in rodents. Hence, by increasing the volume or function of thermogenic fat, one can enhance the metabolic benefits of these unique adipocytes. A maximal oxidative capacity of human BAT could be increased by 150% after a cold acclimation (2), while the β3-AR agonist mirabegron could boost human resting metabolic rate by 13% or resting energy expenditure by 10.7% (52, 53). An increased metabolic activity may benefit metabolic diseases, although the contribution of these approaches to weight loss remains either unclear or disappointing. Such outcome may result from the relative lower proportion of BAT in the whole body. However, WAT accounts for 20–35% of the body weight (70). In the case that beige fat exists in WAT contributing to energy consumption (12), it is promising to induce beige fat development in WAT. Notably, in recent years, studies using rodent models have shown a high plasticity of beige adipocytes regarding to its origin and regulation, the results of which have been summarized in our latest review article (71). Unlike BAT, the origin of murine beige adipocytes reported so far could be white adipocyte via transdifferentiation or distinct progenitors including PDGFRα^+^, mural, or MyoD^+^ progenitors via differentiation (72–77). Moreover, the regulatory mechanisms of beige fat development in rodents vary under different circumstances, which also include non-UCP1 dependent and non-β3-AR dependent mechanisms (44, 58, 77–81). Limited knowledge is known about the origin and regulatory mechanisms of adult human BAT. It is believed that the unique adult human BAT could also be heterogeneous, which requires further investigations. The current findings in rodents could provide more evidences and increase possibilities for targeting thermogenic fat to treat obesity and its related metabolic diseases in humans in the future.

## Author Contributions

RP and YC wrote the manuscript. RP, XZ, PM, and YC edited the manuscript and approved the submitted version.

## Conflict of Interest

The authors declare that the research was conducted in the absence of any commercial or financial relationships that could be construed as a potential conflict of interest.
